# Analytic pipelines to assess the relationship between immune response and germline genetics in human tumors

**DOI:** 10.1016/j.xpro.2022.101809

**Published:** 2022-12-14

**Authors:** Rosalyn W. Sayaman, Mohamad Saad, Carolina Heimann, Donglei Hu, Khalid Kunji, Jessica Roelands, Denise M. Wolf, Scott Huntsman, Michele Ceccarelli, Vésteinn Thorsson, Elad Ziv, Davide Bedognetti

**Affiliations:** 1 Department of Laboratory Medicine, Helen Diller Family Comprehensive Cancer Center, University of California, San Francisco, San Francisco, CA 94143, USA; 2 Department of Population Sciences, Beckman Research Institute, City of Hope Comprehensive Cancer Center, Duarte, CA 91010, USA; 3 Biological Sciences and Engineering Division, Lawrence Berkeley National Laboratory, Berkeley, CA 94720, USA; 4 Qatar Computing Research Institute, Hamad Bin Khalifa University, Doha, Qatar; 5 Neuroscience Research Center, Faculty of Medical Sciences, Lebanese University, Beirut, Lebanon; 6 Institute for Systems Biology, Seattle, WA 98109, USA; 7 Department of Medicine, Institute for Human Genetics, Helen Diller Family Comprehensive Cancer Center, University of California, San Francisco, San Francisco, CA 94143, USA; 8 Human Immunology Department, Cancer Program, Research Branch, Sidra Medicine, PO Box 26999, Doha, Qatar; 9 Department of Pathology, Leiden University Medical Center, Leiden, the Netherlands; 10 Department of Electrical Engineering and Information Technology, University of Naples "Federico II", 80128 Naples, Italy; 11 BIOGEM Institute of Molecular Biology and Genetics, 83031 Ariano Irpino, Italy; 12 College of Health and Life Sciences, Hamad Bin Khalifa University, Doha, Qatar; 13 Department of Internal Medicine and Medical Specialties (Di.M.I.), University of Genoa, 16132 Genoa, Italy

**Keywords:** Bioinformatics, Cancer, Genetics, Genomics, Immunology, Gene expression

## Abstract

Germline genetic variants modulate human immune response. We present analytical pipelines for assessing the contribution of hosts’ genetic background to the immune landscape of solid tumors using harmonized data from more than 9,000 patients in The Cancer Genome Atlas (TCGA). These include protocols for heritability, genome-wide association studies (GWAS), colocalization, and rare variant analyses. These workflows are developed around the structure of TCGA but can be adapted to explore other repositories or in the context of cancer immunotherapy.

For complete details on the use and execution of this protocol, please refer to Sayaman et al. (2021).

## Highlights

• Pipelines for assessing the contribution of germline genetics on tumor immune contexture
• Workflow for data download, processing, assembly, curation, and annotation
• Protocols for heritability, GWAS, colocalization, and rare variant analysis
• Visualization tools for exploration of the results by iAtlas and PheWeb

## Abstract (editor highlights)

Publisher’s note: Undertaking any experimental protocol requires adherence to local institutional guidelines for laboratory safety and ethics.

## Graphical abstract

**Figure:**
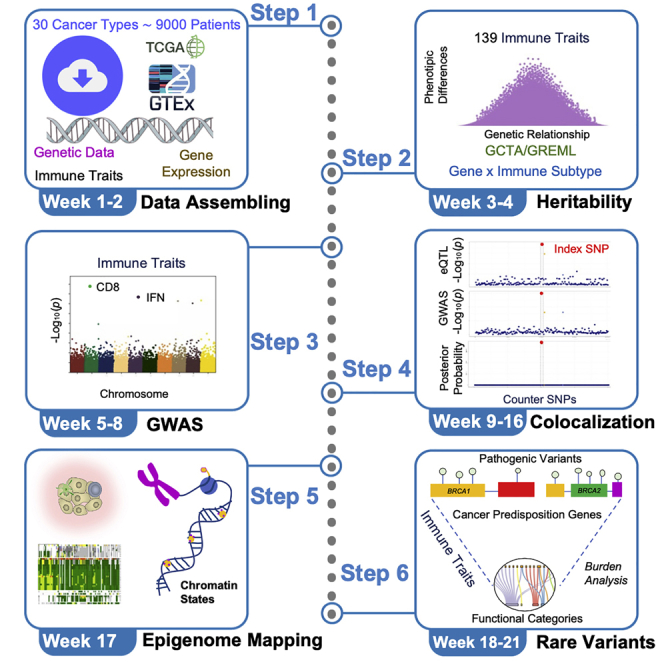

## Teaser

Germline genetic variants modulate human immune response. We present analytical pipelines for assessing the contribution of hosts’ genetic background to the immune landscape of solid tumors using harmonized data from more than 9,000 patients in The Cancer Genome Atlas (TCGA). These include protocols for heritability, genome-wide association studies (GWAS), colocalization, and rare variants analysis. These workflows are developed around the structure of TCGA but can be adapted to explore other repositories or in the context of cancer immunotherapy.

## Before you begin

These protocols describe specific bioinformatic workflows for the analyses of The Cancer Genome Atlas (TCGA) genomic datasets and well-characterized immune traits (Thorsson et al., 2018). However, these methodologies can also be applied to other datasets with similar structures. The majority of analyzed immune traits are derived from gene expression data and can be adapted to other studies.

### Institutional permissions

**Timing: 1–3 weeks (for step 1)**

Authorization from the database of Genotypes and Phenotypes (dbGaP) is necessary to access TCGA germline genetic data (whole exome sequencing (WES) and single nucleotide polymorphism (SNP) array data, including derived imputed SNP data) and Genotype-Tissue Expression (GTEx) genotype data. In addition, download of GTEx summary statistics from the GTEx Google Cloud bucket is a requester-paid download service (see more details in the “before you begin: download datasets” section). GTEx genotype data and summary statistics are only required for colocalization and expression/splicing quantitative trait locus (eQTL/sQTL) analyses.

1. To apply for dbGAP, an institutional account is required.
2. Apply for dbGaP authorization to access TCGA and GTEx controlled access data: https://dbgap.ncbi.nlm.nih.gov/aa/wga.cgi?page=login.
3. Prepare a data access request: https://www.ncbi.nlm.nih.gov/projects/gap/cgi-bin/GetPdf.cgi?document_name=GeneralAAInstructions.pdf.

**CRITICAL:** Preparing the application is fast, however, the review process of the application can take few weeks and should be considered ahead of time.

### Software installation

**Timing: 1 day**

4. PLINK installation.
  a. Install PLINK (1.9 or current version) (Chang et al., 2015).
  b. Download and software documentation is available at: https://www.cog-genomics.org/plink2.
5. bcftools installation.
  a. Install bcftools (1.9 or current version) (Danecek et al., 2021).
  b. Download and software documentation is available at: https://samtools.github.io/bcftools/.
6. Genome-wide Complex Trait Analysis (GCTA) software package installation.
  a. Install GCTA (1.91.2beta or current version) (Yang et al., 2011).
  b. Download and software documentation is available at: https://cnsgenomics.com/software/gcta.
7. R/Bioconductor and related packages installation.
  a. Install R (3.5.0 or current version). Download and software documentation is available at: https://www.r-project.org/.
  b. Install Bioconductor (3.7 or current version) (Huber et al., 2015). Installation instructions and documentation is available at: https://www.bioconductor.org/.
  c. Install the R package: snplist (0.18.1 or current version) (Yi et al., 2017). Installation instructions and documentation is available at: https://cran.r-project.org/web/packages/snplist/index.html.
  d. Install the Bioconductor package: SNPlocs.Hsapiens.dbSNP144.GRCh37 (0.99.20 or current version) (Pagès, 2017). Installation instructions and documentation is available at: https://bioconductor.org/packages/release/data/annotation/html/SNPlocs.Hsapiens.dbSNP144.GRCh37.html.
  e. Install the Bioconductor package: biomaRt (2.36.1 or current version) (Durinck et al., 2005) (Durinck et al., 2009). Use host: grch37.ensembl.org. Installation instructions and documentation is available at: https://bioconductor.org/packages/release/bioc/html/biomaRt.html.
  f. Install the Bioconductor package: GenomicRanges (1.32.7 or current version) (Lawrence et al., 2013). Installation instructions and documentation is available at: https://bioconductor.org/packages/release/bioc/html/GenomicRanges.html.
  g. Install the Bioconductor package: rtracklayer (1.40.6 or current version) (Lawrence et al., 2009). Installation instructions and documentation is available at: https://bioconductor.org/packages/release/bioc/html/rtracklayer.html.
  h. Install the Bioconductor package: AnnotationHub (2.12.1 or current version) (Morgan, 2018). Installation instructions and documentation is available at: https://bioconductor.org/packages/release/bioc/html/AnnotationHub.html.
  i. Install the Bioconductor package: EnsDb.Hsapiens.v86 (2.99.0 or current version) (Rainer, 2017). Installation instructions and documentation is available at: https://bioconductor.org/packages/release/data/annotation/html/EnsDb.Hsapiens.v86.html.
8. LocusZoom installation.
  a. Install LocusZoom (Genome Build/LD Population: hg19/100 Genomes Nov 2014 EUR) (Pruim et al., 2010) from http://locuszoom.org/.
9. eCAVIAR installation.
  a. eCAVIAR (Hormozdiari et al., 2016) from http://zarlab.cs.ucla.edu/tag/ecaviar/.

***Note:*** Indicated software and package versions were used as described in (Sayaman et al., 2021). If using other or future versions of the software and packages, please check for compatibility and version-specific default parameters.

Additional notes regarding the selection and usage of the indicated software are provided below.

*PLINK:* Used for GWAS Analysis. PLINK is a commonly used software for large-scale genetic analyses such as GWAS. It consists in a fast implementation of linear and logistic regression and should produce same results as any implementation of those regression methods.

*bcftools:* BCFtools is a set of utilities that manipulate variant calls in the Variant Call Format (VCF) and its binary counterpart BCF.

*GCTA*: Used for heritability analysis. GCTA is one of first and well-established software packages for estimation of the proportion of phenotypic variance explained by all genome-wide SNPs for a complex trait (Yang et al., 2011). However, this is an area of active of research and newer methods are being developed.

*R/Bioconductor:* Used for TCGA eQTL, rare variant analyses, annotation, immune trait correlation and other analyses as indicated in the manuscript (Sayaman et al., 2021). R/Bioconductor packages offer standard toolsets that offer genomic annotation and genomic mapping functionality. New packages are released and updated quickly by an active community. Alternate software packages that provide similar functionality could be used but may vary based on curation and version of annotation databases.

*LocusZoom:* Used to visualize SNP location in the context of GWAS results. LocusZoom is commonly used and user friendly. It has online and standalone versions. Standalone version (used here) allows us to integrate various sources of Linkage Disequilibrium (LD).

*eCAVIAR:* Used for colocalization analysis. eCAVIAR is a commonly used method for colocalization analyses, and, as compared with other methods, has the advantage of modeling the LD (Hormozdiari et al., 2016).

### Download datasets

**Timing: 1–2 days**

10. Download TCGA Sample List.
  a. Download “Table S1. TCGA Sample List” used in the analysis from (Sayaman et al., 2021).
  b. Read the “Column Headers” sheet descriptor as a guide.
11. Download TCGA Immune Traits.
  a. Download “Table S2. Annotations and Values of Immune Traits” from (Sayaman et al., 2021).
  b. Read the “Column Headers” sheet descriptor as a guide.
12. Download TCGA genotyping data.
  a. The TCGA quality-controlled genotyping data was imputed to the Haplotype Reference Consortium (HRC) panel (McCarthy et al., 2016). These data were generated in (Sayaman et al., 2021) and contributed towards ancestry analyses in (Carrot-Zhang et al., 2020) and are accessible at the associated Genomic Data Commons (GDC) publication page (https://gdc.cancer.gov/about-data/publications/CCG-AIM-2020).
  b. Download the open-access files from the “TCGA QC HRC Imputed Genotyping Data used by the AIM AWG from (Sayaman et al., 2021)” section of the “Supplemental Data Files”:
    i. Information on composition of genotyping files:
      “READ_ME.txt”.
    ii. File mapping of TCGA Patient ID to corresponding Birdseed genotyping files:
      “Map_TCGAPatientID_BirdseedFileID.txt”.
    iii. QC Unimputed Genotyping Data:
      “READ_ME_1.txt” (Read me for quality-controlled unimputed genotyping data).
    iv. HRC Imputed Genotyping Data:
      “READ_ME_4.txt” (Read me for quality-controlled HRC imputed genotyping data).
  c. Download the controlled access files from the “TCGA QC HRC Imputed Genotyping Data used by the AIM AWG from (Sayaman et al., 2021)” section of the “Supplemental Data Files”:
    i. To download the controlled access data, follow instructions under the “Instructions for Data Download” for “Controlled Access Data”. The necessary manifest files are found under the “Data in the GDC” section for “Controlled Access Data”.
    ii. Download the following “QC Unimputed Genotyping Data” files:
      “QC_Unimputed_plink.zip” (Quality-controlled unimputed genotyping data plink files).
    iii. Download the following “HRC Imputed Genotyping Data” files:
      “HRC imputed genotyping data for chromosome 1 - chr_1.zip”.
      “HRC imputed genotyping data for chromosome 2 - chr_2.zip”.
      “HRC imputed genotyping data for chromosome 3 - chr_3.zip”.
      “HRC imputed genotyping data for chromosome 4 - chr_4.zip”.
      “HRC imputed genotyping data for chromosome 5 - chr_5.zip”.
      “HRC imputed genotyping data for chromosome 6 - chr_6.zip”.
      “HRC imputed genotyping data for chromosome 7 - chr_7.zip”.
      “HRC imputed genotyping data for chromosome 8 - chr_8.zip”.
      “HRC imputed genotyping data for chromosome 9 - chr_9.zip”.
      “HRC imputed genotyping data for chromosome 10 - chr_10.zip”.
      “HRC imputed genotyping data for chromosome 11 - chr_11.zip”.
      “HRC imputed genotyping data for chromosome 12 - chr_12.zip”.
      “HRC imputed genotyping data for chromosome 13 - chr_13.zip”.
      “HRC imputed genotyping data for chromosome 14 - chr_14.zip”.
      “HRC imputed genotyping data for chromosome 15 - chr_15.zip”.
      “HRC imputed genotyping data for chromosome 16 - chr_16.zip”.
      “HRC imputed genotyping data for chromosome 17 - chr_17.zip”.
      “HRC imputed genotyping data for chromosome 18 - chr_18.zip”.
      “HRC imputed genotyping data for chromosome 19 - chr_19.zip”.
      “HRC imputed genotyping data for chromosome 20 - chr_20.zip”.
      “HRC imputed genotyping data for chromosome 21 - chr_21.zip”.
      “HRC imputed genotyping data for chromosome 22 - chr_22.zip”.
      ***Note:*** Please cite (Sayaman et al., 2021) when using the quality-controlled and HRC imputed genotyping data.
      **CRITICAL:** Access to the TCGA quality-controlled and HRC imputed genotyping data generated requires dbGaP controlled access permission approval. See before you begin: Apply for dbGaP Authorization.
      ***Note:*** The protocol for generating the TCGA HRC Imputed Genotyping Data from raw TCGA birdseed Affymetrix SNP 6.0 array is discussed in (Chambwe et al., 2022) and supplemental code is made available at: https://github.com/rwsayaman/TCGA_PanCancer_Genotyping_Imputation.
13. Download TCGA whole exome sequencing data.
  a. Download WES data file from https://gdc.cancer.gov/about-data/publications/PanCanAtlas-Germline-AWG:
    “PCA.r1.TCGAbarcode.merge.tnSwapCorrected.10389.vcf.gz”.

**CRITICAL:** Access to the TCGA whole exome sequencing data requires dbGaP controlled access permission approval. Before you begin: Apply for dbGaP Authorization.

14. Download GTEx data.
  a. Information regarding GTEx data are available at the GTEx Portal: https://gtexportal.org/.
  b. Under “Datasets” access the “Data Download” site to access QTL summary statistics. Check the current release version.
    i. Under “Single-Tissue cis-QTL Data”, click on the link and follow instructions for downloading all SNP-gene associations from the GTEx Google Cloud bucket:
      “eQTL Tissue-Specific ALL SNP Gene Association”.
      “sQTL Tissue-Specific ALL SNP Gene Association”.
    ii. From the “GTEx_Analysis_v8_QTLs” folder, download all files from the following folders:
      “GTEx_Analysis_v8_eQTL_all_associations”.
      “GTEx_Analysis_v8_sQTL_all_associations”.
  c. Under “Datasets” access the “Protected Data” site and follow instructions to gain access to GTEx v8 genotyping data hosted in the AnVIL repository.
    ***Note:*** GTEx Release v8 was used in (Sayaman et al., 2021). Download from GTEx Google Cloud bucket is a requester-paid download service. Create a Google Cloud account with sufficient funds to download. Set-up the appropriate billing project.
    **CRITICAL:** Access to the GTEx genotyping data requires dbGaP controlled access permission approval. See before you begin: Apply for dbGaP Authorization.
15. Download the Ensembl Variant Effect Predictor (VEP) annotation.
  a. Input file, “vep_input.txt”, is a text file that contains eight columns, space or tab-delimited: Chromosome, Position, Uploaded_Variation, ReferenceAllele, AlternativeAllele, dummy1, dummy2, dummy3. Dummy columns are not needed and were filled with ‘.’
  b. Upload “vep_input.txt” to https://grch37.ensembl.org/Tools/VEP. Select list of annotations to extract and then run. When the job is done, download output text (.txt) format, which is more suitable for Excel.
  c. Combine annotations per SNP. VEP provides multiple annotations per SNP. SNPs might map to different genes and could have several biological impacts on nearby genes. Use R script “CombineVEPannotations.R” to combine annotations.
  d. Example of combined annotations: “CXXC5:synonymous_variant:LOW|CXXC5:upstream_gene_variant:MODIFIER”.
16. Download Roadmap Epigenomics Project Epigenomic State Model.
  a. Access the Expanded 18-state model (6 marks, 98 epigenomes) for Build GRCh37/hg19: https://egg2.wustl.edu/roadmap/web_portal/chr_state_learning.html#exp_18state.
  b. Under “MNEMONICS BED FILES” section, download the archive of all mnemonics bed files: “all.mnemonics.bedFiles.tgz”.

### Download GitHub repository

**Timing: 5 min**

The scripts and code descriptions used in the entirety of this protocol are available at:

https://github.com/rwsayaman/TCGA_PanCancer_Immune_Genetics.

17. Download or clone the GitHub repository: “TCGA_PanCancer_Immune_Genetics” (Sayaman et al., 2021).
  a. Review all “README” files for each section of workflow.
  b. Ensure all the necessary code has been downloaded.

***Optional:*** This protocol is designed to work with pre-processed and quality-controlled genotyping data. If users start from raw genotyping data from SNP arrays, please see the companion protocol for quality-control analysis of germline data, stranding and genotype imputation from (Chambwe et al., 2022) and the associated scripts and code descriptions at: https://github.com/rwsayaman/TCGA_PanCancer_Genotyping_Imputation.

18. Download or clone the GitHub repository: “TCGA_PanCancer_Genotyping_Imputation” (Chambwe et al., 2022).
  a. Review all “README” files for each section of workflow.
  b. Ensure all the necessary code has been downloaded.

***Note:*** The example code provided were designed to run on specific high-performance compute environment. Users should adjust the code to match the capabilities of their compute environment and queuing system.

For reference, analyses carried out using the software listed above are described in the following sections in the manuscript were performed on multiple severs including (1) the University of California, San Francisco (UCSF) Wynton high-performance (HPC) cluster, which currently contains 449 nodes with over 12572 Intel CPU cores and 42 nodes containing a total of 148 NVIDIA GPUs, (2) the original UCSF TIPCC HPC cluster (now C4), which had 8 communal compute nodes and 1 dedicated node, each with 12–64 cores (each node had from 64 to 512 GiB of RAM and at least 1.8 TiB of fast local disk space), and (3) two additional severs with 32 and 48 CPUs (Intel(R) Xeon(R) CPU E5-2650 0 @ 2.00 GHz), respectively. In the optimization phase, analyses have been performed by different operators and at multiple times to ensure accuracies and reproducibility. As these are shared servers, time might considerably vary in function of the number of nodes available. From a computational point of view, the most time-consuming step is the GWAS (3–4 h for each trait, as average). However, file preparation, as well as annotation and curation of the output files is particularly time consuming. An estimated time, considering these manual steps and factoring in some troubleshooting time (see also troubleshooting section) is indicated for each component in the following sections. In terms of file preparation, the GTEx colocalization might be the most time-consuming component.

### Pre-processing of imputed data

**Timing: 1–2 days**

This section describes the pre-processing steps necessary to use the HRC Imputed Genotyping Data we generated as a general resource for this specific analysis (Sayaman et al., 2021).

19. Unzip the individual HRC imputed genotyping data for each chromosome (chr_∗.zip).
  a. Read the downloaded “READ_ME∗.txt” files associated with HRC imputed genotyping data from before you begin: download datasets.
  b. Zipped data is password protected, see “READ_ME_4.txt.”
    See code to unzip: “qsub_unzip_HRC_chr.txt.”
  c. Each chr unzipped folder contains 3 files:
    “dose.vcf.gz” - imputed genotypes with dosage information.
    “dose.vcf.gz.tbi” - index file of the .vcf.gz file.
    “.info.gz” file - information for each variant including quality and frequency.
    ***Note:*** For Michigan Imputation Server Minimac3 output file information, see: https://genome.sph.umich.edu/wiki/Minimac3_Info_File.
20. For each chromosome, filter the HRC imputed genotyping data to exclude SNPs with imputation R^2^ < 0.5. The imputation R^2^ is the estimated value of the squared correlation between imputed genotypes and true, unobserved genotypes.
  a. Filter each “chr∗.dose.vcf.gz” files and generate “chr∗.rsq0.5.dose.vcf.gz” imputed genotype files of SNPs with R^2^ ≥ 0.5.
  b. Index and generate the corresponding “chr∗.rsq0.5.dose.vcf.gz.tbi” files.
  c. Generate the corresponding filtered “chr∗.info.rsq0.5.gz” information files.
21. For each chromosome, filter HRC imputed genotyping data to exclude SNPs with minor allele frequency (MAF) < 0.005.
  a. Convert VCF “chr∗.rsq0.5.dose.vcf.gz” files to PLINK “tcga_imputed_hrc1.1_rsq0.5_chr∗.bed” files.
  b. **Optional:** Rename SNP ID names in the HRC imputed dataset with alleles listed in alphabetical order to assist matching with other datasets.
    ***Note:*** This only affects the SNP ID name and not the encoding of the A1 and A2 alleles in PLINK which is maintained.
  c. **Optional:** In PLINK, exclude SNPs with MAF < 0.005 (--maf).
    ***Note:*** Minor allele frequency (MAF) filtering can be performed at this step to reduce the HRC imputed genotyping data input file size. Alternatively, GWAS analysis can be performed using the R^2^ filtered-HRC imputed genotyping data from “before you begin: Pre-processing of Imputed Data” step 20 – e.g., as in (Sayaman et al., 2021), while MAF filtering can be performed on the summary statistics using the recalculated MAF from included subjects (see “step-by-step method details: genome-wide association study (GWAS)” steps 16, 23b).
    ***Note:*** Scripts and code description used in this section are available at: https://github.com/rwsayaman/TCGA_PanCancer_Immune_Genetics.

Direct link: Pre-processing of Imputed Data. The example code in this section were optimized for the high-performance compute environment at UCSF HPC employing Portable Batch System (PBS) job scheduling; consult your system administrator to adapt the provided code to your system.

## Key resources table

**Table:** 

REAGENT or RESOURCE	SOURCE	IDENTIFIER
**Biological samples**		

Tumor Samples	Primary tumor samples, TCGA	See “Table S1. TCGA Sample List” used in the analysis from (Sayaman et al., 2021) for TCGA identifiers of biological samples included.
Normal Samples	Whole blood or surrounding normal tissue, TCGA	See “Table S1. TCGA Sample List” used in the analysis from (Sayaman et al., 2021) for TCGA identifiers of biological samples included.

**Deposited data**		

Germline Genotype Data	TCGA Affymetrix 6.0 array genotype type processed via Birdseed	https://portal.gdc.cancer.gov/
Germline Genotype Data	TCGA HRC Imputed Germline Genotype Data (SNPs) (Sayaman et al., 2021) (Carrot-Zhang et al., 2020)	https://gdc.cancer.gov/about-data/publications/CCG-AIM-2020
Germline Genotype Data	TCGA Whole exome sequencing data (Huang et al., 2018)	https://portal.gdc.cancer.gov/
Gene expression data	TCGA RNA-sequencing data	https://portal.gdc.cancer.gov/
MANTIS score	(Bonneville et al., 2017)	https://github.com/OSU-SRLab/MANTIS
Immune traits	(Thorsson et al., 2018)	https://pubmed.ncbi.nlm.nih.gov/29628290/
List of samples to be used in the analysis: TCGA Exome and Genotype VCF Barcodes, TCGA Birdseed barcodes; Covariates: Genetic Ancestry PC1-7, Sex, Age, and Cancer Type	Table S1 from (Sayaman et al., 2021)	https://pubmed.ncbi.nlm.nih.gov/33567262/
Selection and values of non redundant immune traits used for the current the analyses	Table S2 from (Sayaman et al., 2021)	https://pubmed.ncbi.nlm.nih.gov/33567262/
Haplotype reference consortium (version r1.1.2016)	(McCarthy et al., 2016)	http://www.haplotype-reference-consortium.org/
SNP annotations	Ensembl Variant Effect Predictor (VEP)	https://grch37.ensembl.org/info/docs/tools/vep/index.html
GTEx Version 8 QTL summary statistics	GTEx Portal	https://www.gtexportal.org
GTEx Version 8 genotypes	dbGAP	https://www.ncbi.nlm.nih.gov/projects/gap/cgi-bin/study.cgi?study_id=phs000424.v8.p2
TCGA Splicing data	Percent Spliced In (PSI)	https://portal.gdc.cancer.gov/
DICE	Database of Immune Cell Expression, Expression quantitative trait loci (eQTLs) and Epigenomics	https://dice-database.org

**Software and algorithms**		

PLINK 1.9	(Chang et al., 2015)	http://zzz.bwh.harvard.edu/plink/ https://www.cog-genomics.org/plink/
Minimac3 (HRC r1.1.2016 reference panel)	(Howie et al., 2012) (Fuchsberger et al., 2015)	https://genome.sph.umich.edu/wiki/Minimac3
bcftools 1.9	(Danecek et al., 2021)	https://samtools.github.io/bcftools/
GCTA GREML 1.91.2beta	(Yang et al., 2011)	https://cnsgenomics.com/software/gcta/#Download
R 3.5.0	The R Project for Statistical Computing	https://www.r-project.org/
R package: snplist_0.18.1	(Yi et al., 2017)	https://cran.r-project.org/web/packages/snplist/index.html
Bioconductor 3.7	(Huber et al., 2015)	https://www.bioconductor.org/
Bioconductor package: SNPlocs.Hsapiens.dbSNP144.GRCh37_0.99.20	(Pagès, 2017)	https://bioconductor.org/packages/release/data/annotation/html/SNPlocs.Hsapiens.dbSNP144.GRCh37.html
Bioconductor package: biomaRt_2.36.1 (Host: grch37.ensembl.org)	(Durinck et al., 2005), (Durinck et al., 2009)	https://bioconductor.org/packages/release/bioc/html/biomaRt.html
Bioconductor package: GenomicRanges_1.32.7	(Lawrence et al., 2013)	https://bioconductor.org/packages/release/bioc/html/GenomicRanges.html
Bioconductor package: rtracklayer_1.40.6	(Lawrence et al., 2009)	https://bioconductor.org/packages/release/bioc/html/rtracklayer.html
Bioconductor package: AnnotationHub_2.12.1	(Morgan, 2018)	https://bioconductor.org/packages/release/bioc/html/AnnotationHub.html
Bioconductor package: EnsDb.Hsapiens.v86_2.99.0	(Rainer, 2017)	https://bioconductor.org/packages/release/data/annotation/html/EnsDb.Hsapiens.v86.html
LocusZoom (Genome Build/LD Population: hg19/100 Genomes Nov 2014 EUR)	(Pruim et al., 2010)	http://locuszoom.org/
eCAVIAR	(Hormozdiari et al., 2016)	http://zarlab.cs.ucla.edu/tag/ecaviar/
Python 2.7.5	Python Software Foundation	https://www.python.org/downloads/release/python-275/

**Other**		

Resource website and custom scripts	This paper, (Sayaman et al., 2021), (Chambwe et al., 2022)	https://github.com/rwsayaman/TCGA_PanCancer_Immune_Genetics https://github.com/rwsayaman/TCGA_PanCancer_Genotyping_Imputation

***Note:*** “Table S1” and “Table S2” in the “key resources table” refers to supplementary tables from (Sayaman et al., 2021).

## Materials and equipment

The overall disk space needed for the project is 2.67 TB. Breakdown for different data types and analyses are provided below (Table 1).

**Table 1:** Disk space requirements for analytic protocols

	Data (Gb)	Analysis (Gb)
**TCGA**		

Pre-processing (genotyping data)	210	440
Immune Traits (Table S2) (Sayaman et al., 2021)	0.005	0.075
Heritability Analysis (Table S1) (Sayaman et al., 2021)	0.005	55
GWAS (Table S1) (Sayaman et al., 2021)	0.005	155
eQTL (gene expression matrix)	4	8
sQTL (sqtl stats)	95	25
Colocalization	–	25
Rare Variant Analysis (WES)	500	64

**GTEx**		

eQTL (summary stats)	190	30
sQTL (summary stats)	695	
Colocalization (genotyping data)	15	160

**Roadmap epigenomics project**		

Epigenomic Analysis	0.3	0.035

**Table 2:** Summary of figures from the (Sayaman et al., 2021) manuscript that can be reproduced in CRI iAtlas, with a description of available expanded analysis

Figure in Sayaman et al. (2021)	Expanded analysis
2A	Selection of p-value threshold for inclusion of results. Visualization of heritability of a given immune trait, immune trait category or immune trait module across different ancestry clusters.
2B	Selection of p-value threshold for inclusion of results.
3A	Selection of immune features of interest to include or exclude from the Manhattan plot. Zoom to region, gene or SNP of interest. Visualization of genomic regions in IGV tracks. Link of SNP of interest to external databases (dbSNP, GTEx, GWAS Catalog, DICE, PheWeb).
4A, 4C, 4E, 4G	Visualization of GWAS hits with -log10p(GWAS) > 6. Zoom to region, gene or SNP of interest. Visualization of genomic regions in IGV tracks. Link of SNP of interest to external databases (dbSNP, GTEx, GWAS Catalog, DICE, PheWeb).
5A	Selection of immune features of interest to include or exclude from the Manhattan plot. Zoom to region, gene or SNP of interest. Visualization of genomic regions in IGV tracks. Link of SNP of interest to external databases (dbSNP, GTEx, GWAS Catalog, DICE, PheWeb).
5C	Visualization of GWAS hits with -log10p(GWAS) > 6. Zoom to region, gene or SNP of interest. Visualization of genomic regions in IGV tracks. Link of SNP of interest to external databases (dbSNP, GTEx, GWAS Catalog, DICE, PheWeb).
5D	No expanded analysis available
6B	Selection of functional pathway of interest. Summary statistics available for download.

## Step-by-step method details

### Immune traits

**Timing: 1 day**

139 immune traits used in the analyses were curated from (Thorsson et al., 2018) by selecting non-redundant traits. Immune trait categories were defined based on the methodological source of the data. Immune trait functional modules were defined based on Pearson correlation analysis.

1. Prepare curated set immune traits genes.
  a. Curated set of 139 immune traits in TCGA can be downloaded from (Sayaman et al., 2021) “Table S2”.
  b. For calculation of immune traits in a new dataset, consult (Thorsson et al., 2018) methods section describing methodologies and source papers for derivation of specific immune traits.
    i. Code to generate gene expression signatures from (Wolf et al., 2014) (Amara et al., 2017) and single sample gene set enrichment (ssGSEA) signatures from (Bindea et al., 2013; Şenbabaoğlu et al., 2016) are provided as a resource in the (Sayaman et al., 2021) GitHub repository (see link in the note below).
    ii. Necessary transformation of immune trait values in TCGA for use in genetic analysis described below are annotated in (Sayaman et al., 2021) “Table S2”.
2. Calculate Pearson correlations of continuous values of the 139 immune traits.
3. Generate a correlation heatmap with a hierarchical clustering dendrogram.
  a. Cluster correlation matrix using complete agglomerative hierarchical clustering method and (1-correlation) as distance metric.
4. Identify highly correlated blocks (dendrogram clusters) of immune traits to generate immune functional modules.

***Note:*** Scripts and code description used in this section are available at: https://github.com/rwsayaman/TCGA_PanCancer_Immune_Genetics.

Direct link: Immune Traits.

### Heritability analysis

**Timing: 2 weeks**

Heritability analysis on 139 traits is conducted using a mixed-model approach implemented in the genome-wide complex trait analysis (GCTA) software package with the genomic-relatedness-based restricted maximum-likelihood (GREML) method (Yang et al., 2010) (Yang et al., 2011). This calculates the proportion of immune trait variation that is attributable to common genetic variants (% Heritability). Refer to the GCTA website for details (https://cnsgenomics.com/software/gcta).

5. Format immune trait input file:
  a. Formatted input file of TCGA immune traits is provided in the (Sayaman et al., 2021) GitHub repository:

“Immune.pheno.139.source.coded.TCGAID.9769.txt”.

6. Identify genetic ancestry assignment of each individual and create a filtered sample list for each genetic ancestry cluster:
  a. Ancestry assignments for each TCGA individual are provided in “Table S1. TCGA Sample List Used in the Analysis” from (Sayaman et al., 2021).
  b. Formatted input file of TCGA patient barcodes assigned to each genetic ancestry cluster is provided in the (Sayaman et al., 2021) GitHub repository:

“TCGAID_Cluster1.EUR.8036.txt”

“TCGAID_Cluster2.ASIAN.605.txt”

“TCGAID_Cluster3.AFR.904.txt”

“TCGAID_Cluster4.AMR.222.txt”

7. To conduct heritability analyses within each ancestry subgroup (N_European_=7,813, N_African_=863, N_Asian_=570, and N_American_=209 individuals), subset individuals belonging to specified ancestry group from the QC TCGA HRC imputed genotyping data in PLINK (--keep) using ancestry assignments.

plink --bfile [input_file]

--keep [ancestry_cluster_sample_file_list]

--make-bed

--out [output_filename]

See script:

“qsub_plink_whitelist_geno_mind_unique.indv_chr.auto_hardy.nonriskSNP_maf_uniqueSNP_TCGAID_ancestry.txt”.

***Note:*** Analysis is automatically performed on samples with complete data. A subset of the defined samples within each genetic ancestry cluster are automatically skipped due to missing data (e.g., immune trait or covariate values).

8. Estimate the genetic relatedness matrix (GRM) from all the autosomal SNPs with MAF > 0.01 within each ancestry group using GCTA:

gcta64 --bfile [input_filename]

--autosome

--maf 0.01

--make-grm

--out [output_filename]

--thread-num [numeric_value_number_threads]

See script:

“qsub_gcta_whitelist_geno_mind_unique.indv_chr.auto_hardy.nonriskSNP_maf_uniqueSNP_TCGAID_ancestry_grm.txt”.

9. Filter out individuals for relatedness. GCTA removes one of a pair of individuals with estimated relatedness larger than the specified cut-off value (cut-off = 0.05):

gcta64 --grm [input_filename]

--grm-cutoff 0.05

--make-grm

--out [output_filename]

See script:

“qsub_gcta_whitelist_geno_mind_unique.indv_chr.auto_hardy.nonriskSNP_maf_uniqueSNP_TCGAID_ancestry_grm_grm.cutoff.0.05.txt”.

10. Run GCTA GREML unconstrained (using default algorithm: Average Information) to estimate variance explained by SNPs with defined categorical and continuous covariates using the following parameters:

gcta64 --reml-no-constrain

--grm [input_filename]

--pheno [immune_trait_matrix_filename]

--mpheno [immune_trait_numeric_index_input_matrix]

--covar [categorical_covariates_filename]

--qcovar [continuous_covariates_filename]

--thread-num [numeric_value_number_threads]

--out [output_filename]

See script: “qsub_grm.cutoff.0.05_greml_EUR.ImmunePheno216_CancerTypeSex.covar_PCA.AgeYears.qcovar.txt”.

**CRITICAL:** Run heritability analysis unconstrained. This will produce heritability estimates (Vg/Vp) and standard deviations outside the 0–1 range.

11. From GCTA GREML “.hsq” result file, extract the ratio of genetic variance to phenotypic variance (Vg/Vp), estimate and SE; the LRT p-value and sample size (n) for each immune trait.
12. Concatenate heritability analysis results across all immune traits tested.
  a. Annotate each result file with the corresponding immune trait, immune category and immune module (see “Table S2”, (Sayaman et al., 2021)).
  b. Append annotated result files from each immune trait.
13. Correct for multiple-hypothesis testing ancestry group by calculating the FDR p-value using the Benjamini-Hochberg adjustment method.
14. **Optional:** Visualize % heritability (Vg/Vp ∗ 100) across all immune traits per ancestry group for exploratory data analysis.

***Note:*** Scripts used in this section are available at: https://github.com/rwsayaman/TCGA_PanCancer_Immune_Genetics.

Direct link: Heritability Analysis. The example code in this section were optimized for the high-performance compute environment at UCSF HPC employing Portable Batch System (PBS) job scheduling; consult your system administrator to adapt the provided code to your system.

Interactive visualization of heritability analysis from (Sayaman et al., 2021) can be done in CRI iAtlas (https://www.cri-iatlas.org/), in the “Germline Analysis” module (see the “interactive visualization of results” section of “expected outcomes”).

### Genome-Wide Association Study (GWAS)

**Timing: 4 Weeks**

GWAS were performed on traits that we found to have significant heritability since these would be most likely driven by common genetic variants.

15. Perform Identity-by-Descent (IBD) analysis in PLINK in each ancestry cluster (--genome) and filter individuals out for relatedness (pihat < 0.25). This leaves n=9,603 unrelated individuals in the TCGA cohort in the output file:

“GWAS.IBD.ALL.TCGAID.txt”.

16. Recalculate allele frequencies in PLINK for the subset of individuals used in the analysis in PLINK:

plink --bed [input_bed_filename]

--bim [input_bim_filename]

--fam [input_fam_filename]

--allow-no-sex

--keep-allele-order

--keep [GWAS.IBD.ALL.TCGAID.txt]

--freq

--out [Freq/output_filename]

See script: “qsub_plink_freq_GWAS.IBD.ALL.txt”.

17. Prepare the covariate file:

“covar.GWAS.IBD.ALL.txt”.

18. Prepare the phenotype file:

“Immune.phenotype.33.Set∗.GWAS.txt”.

19. Run linear association analysis for each continuous immune traits in PLINK:

plink --bed [input_bed_filename]

--bim [input_bim_filename]

--fam [input_fam_filename]

--allow-no-sex

--keep-allele-order

--keep [GWAS.IBD.ALL.TCGAID.txt]

--pheno [Immune.phenotype.33.Set∗.GWAS.txt]

--all-pheno

--covar [covar.GWAS.IBD.ALL.txt]

--linear hide-covar

--out [output_filename]

See script: “qsub_plink_linear_GWAS.IBD.ALL_Immune.33.Wolf.Set1.txt”.

20. Run logistic regression on dichotomized discrete immune traits in PLINK by modifying the code in step 19 using the logistic command (--logistic).

***Note:*** “.bed”, “.bim” and “.fam” input files are provided separately in the sample code because SNP ID names from the original HRC imputed dataset were renamed with alleles listed in alphabetical order to assist matching with other datasets. This only affects the SNP ID name and not the encoding of the A1 and A2 alleles in PLINK which is maintained.

21. Filter resulting summary statistics from PLINK based on test p-values (P in PLINK). Genome-wide significance was defined at p < 5 × 10^−8^ and suggestive significance at p < 1 × 10^−6^ in our study.

See script:

“r_plotResults_GWAS.IBD.ALL_Immune.33.Set∗.r” and "qsub_r_plotResults_GWAS.IBD.ALL_Immune.33.Set∗.txt".

22. **Optional:** Visualize results for exploratory data analysis:
  a. Manhattan plot, plotting GWAS -log10 p-value against the base pair position per chromosome.
  b. Quantile-quantile plot (Q-Q plot), plotting the quantile distribution of observed p-values for each SNP against expected values from a theoretical χ2-distribution; calculate the genomic inflation factor (lamba), the median of the χ2 test statistics divided by the expected median of the χ2 distribution.

***Note:*** Interactive visualization of GWAS from (Sayaman et al., 2021) can be done in CRI iAtlas (https://www.cri-iatlas.org/), in the “Germline Analysis” module or using the PheWeb tool (https://pheweb-tcga.qcri.org/) (see the “interactive visualization of results” section of “expected outcomes”).

23. **Optional:** Each SNP can be further annotated (see “Table S4”, (Sayaman et al., 2021)) with:
  a. The Minimac3 HRC imputation information for each SNP (extracted from the filtered chr∗.info.rsq0.5.gz), including whether SNP was genotyped or imputed (Genotyped), the estimated value of the squared correlation between imputed genotypes and true, unobserved genotypes (Rsq), the average probability of observing the most likely allele for each haplotype (AvgCall), minor allele frequency of the variant in the imputed data (MAF) (https://genome.sph.umich.edu/wiki/Minimac3_Info_File).
    i. Map the Minimac3 HRC imputation information to the GWAS summary stats using the variant identifier.
      See script: “r_plotResults_GWAS.IBD.ALL_Immune.33.Set∗.r” and "qsub_r_plotResults_GWAS.IBD.ALL_Immune.33.Set∗.txt".
  b. The recalculated minor allele frequency (MAF) for the GWAS study set (n=9,603) calculated from “step-by-step method details” step 16). This is done as the MAF from Minimac3 HRC imputation information is derived from the full TCGA dataset, whereas individuals used in this GWAS study was further filtered for non-hematologic cancers only and with closely related individuals excluded (by Identity-by-Descent (IBD) in “step-by-step method details” step 15).
    i. Map the recalculated MAF to the GWAS summary stats using the variant identifier.
    ii. Filter summary statistics to exclude SNPs with recalculated MAF < 0.005.
      See script: “r_plotResults_GWAS.IBD.ALL_Immune.33.Set∗.r” and "qsub_r_plotResults_GWAS.IBD.ALL_Immune.33.Set∗.txt".
  c. Map the genomic chromosome and base pair position to corresponding SNP rsIDs and IUPAC nucleotide ambiguity codes using the R “SNPlocs.Hsapiens.dbSNP144.GRCh37” package (https://www.rdocumentation.org/packages/BSgenome/versions/1.40.1/topics/SNPlocs-class).
    i. Transform GWAS results chromosome and base pair position into a GRanges object using the R “GenomicRanges” package.
    ii. Define the set of SNPs as the “SNPlocs.Hsapiens.dbSNP144.GRCh37” dataset.
    iii. Overlap the GRanges chromosome and base pair position with the “SNPlocs.Hsapiens.dbSNP144.GRCh37” dataset using the snpsByOverlaps function.
    iv. Merge annotated data with results file.
      See script: “r_annotation_SNP.r".
      ***Note:*** Not all SNPs have corresponding rsIDs.
  d. The nearest genes to SNP of interest (e.g., +/-50 KB, +/-500 KB, +/- 1000 KB) using R “snplist” package to map rsID to gene maps extracted via the R “biomaRt” package using host grch37.ensembl.org (https://uswest.ensembl.org/info/data/biomart/index.html).
    i. Create SNP information table (setSNPTable function) using SNP rsID, chromosome and base pair position.
    ii. Create a gene information table (setGeneTable function) using gene attributes (gene symbol, Ensembl ID, Entrez ID, chromosome, start and end position, strand) extracted from R “biomaRt” package using host grch37.ensembl.org.
    iii. Find overlaps of the SNP and gene information tables, setting the margin (bp) to the desired distance from SNP of interest (makeGeneSet function).
      See script: “r_annotation_SNP.r".
  e. VEP Impact and Symbol annotation from the downloaded VEP annotation file (see download datasets section).
    i. Map the VEP annotation file (“VEP_MS_improved.csv” to the GWAS summary stats using chromosome and base pair position.
      See script: “r_annotation_SNP.r".
24. Concatenate GWAS filtered (and annotated) summary stats across all immune traits tested.
  a. Annotate each summary stat file with the corresponding immune trait, immune category and immune module (see “Table S4”, (Sayaman et al., 2021).
  b. Append annotated summary stat files from each immune trait GWAS.
25. Identify genome-wide significant loci as SNPs within 50 KB region with at least one genome-wide significant SNP (p < 5 × 10^−8^). This excludes the HLA locus on chr 6 which spans ∼3.5 MB.

***Note:*** Scripts and code description used in this section are available at: https://github.com/rwsayaman/TCGA_PanCancer_Immune_Genetics.

Direct link: Genome-Wide Association Study (GWAS). The example code in this section were optimized for the high-performance compute environment at UCSF HPC employing Portable Batch System (PBS) job scheduling; consult your system administrator to adapt the provided code to your system.

### Epigenomic analysis

**Timing: 1–2 days**

This section describes the mapping of genome-wide significant and suggestive SNPs to the Roadmap Epigenomics Project Epigenomic Expanded 18-state model which uses 6 marks across 98 epigenomes: https://egg2.wustl.edu/roadmap/web_portal/chr_state_learning.html#exp_18state.

(Roadmap Epigenomics Consortium et al., 2015).

26. Transform the Immune-Germline GWAS suggestive and genome-wide significant SNPs results table into a GRanges object using the “GenomicRanges” package in R.
  a. Import the GWAS suggestive and genome-wide significant SNP result table into R.
  b. Create a unique data frame of SNP IDs, chromosome and base pair positions.
  c. Convert into GRanges object using the makeGRangesFromDataFrame function in “GenomicRanges” package.
27. Iteratively loop and import each Roadmap Epigenomics Project epigenome, and extract epigenomic states that overlap each of the Germline-Immune SNP chromosome and base pair position.
  a. Using “rtracklayer” package in R, import each epigenome bed file with the annotated Epigenomic Expanded 18-state model.
  b. At each GRanges SNP chromosome and base pair position, extract the corresponding epigenomic state from each epigenome using the mergeByOverlaps function in “GenomicRanges” package.
28. Map epigenome IDs to corresponding epigenome descriptions of source cell types and tissue types using the “Roadmap.metadata.qc.jul2013.csv” annotation file.
29. Annotate epigenetic states with published color schema using “FigureColors.csv” file.
30. Manually curate immune-associated epigenomes via cell type or tissue of origin (see “Table S4, (Sayaman et al., 2021)).

***Note:*** Scripts and code description used in this section are available at: https://github.com/rwsayaman/TCGA_PanCancer_Immune_Genetics.

Direct link: Epigenomic Analysis.

### Expression and splicing quantitative trait locus analysis (eQTLs and sQTL)

**Timing: 1 week**

This section describes eQTL and sQTL analysis in TCGA and GTEx data. For GTEx data, eQTL/sQTL summary statistics across tissues were downloaded from https://gtexportal.org/home/index.html. For the TCGA dataset, the gene expression matrix was downloaded from: https://gdc.cancer.gov/about-data/publications/pancanatlas and analysis was conducted locally.

31. TCGA Analysis.
  a. eQTL.
    i. Download gene expression matrix: “EBPlusPlusAdjustPANCAN_IlluminaHiSeq_RNASeqV2.geneExp.tsv”.
    ii. Download genes’ starts, ends, and types (i.e., protein coding, pseudogenes, miRNA, etc.) from “biomaRt” package from https://grch37.ensembl.org/biomart/martview/. Required columns from the “mart_report.txt” output files are: “Chromosome”, “Start”, “End”, and “Gene type”.
    iii. Transpose the gene expression matrix. This can be run with R script “OrganizeRNAMatrix.R”.
    iv. Run association analysis between genome-wide and suggestive SNPs and genes within 1 MB using a linear regression model accounting for genetic ancestry PC1-7, sex, age, and cancer type (see “Table S1”, (Sayaman et al., 2021)). This can be achieved using the script “eQTLanalysis.R”. This script also runs eQTL analysis for the 30 cancers separately.
    v. Summarize outcome: results will have the following: chromosome, position, gene name, distance from gene, pan-cancer sample size, pan-cancer effect size, pan-cancer p-value, and then the same information repeated for each cancer type.
  b. sQTL.
    i. Download 5′, 3′, exon skipping, intron retention, and mutually exclusive exon splice events data from https://gdc.cancer.gov/about-data/publications/PanCanAtlas-Splicing-2018.
    ii. Organize the data input and format it for SNP-splicing event association analysis. Use “script PrepareData.sh”. This script keeps the genes that mapped to the most significant SNPs (i.e., Suggestive and GW). Example: grep –f ListGenes.txt splice3prime > Data_3prime. It creates two more files that contain TCGA subject IDs, and splicing event IDs and types. It finally runs an R script “Analyze.R” that performs association analysis between SNP genotypes and splicing events using linear regression model accounting for genetic ancestry PC1-7, sex, age, and cancer type (for pan-cancer analysis only).
    iii. Summarize outcome, an output file containing association results as follows: chromosome, position, ensemble ID, gene name, splicing event ID.
32. GTEx Analysis.
  a. Convert all GWAS suggestive and genome-wide significant SNP chromosome and base pair positions from build GRCh37 to build GRCh38 to match GTEx annotation.
    i. Import the GRanges object of SNP chromosome and base pair positions from 13.
    ii. Load the chain file “AH14150” for Homo sapiens rRNA hg19 to hg38 from the “AnnotationHub” package in R.
    iii. Convert SNP chromosome and base pair positions from build GRCh37 to build GRCh38 using the liftOver function in the “rtracklayer” package in R.
      See script: “GWAS.SNPs_liftOver.GRCh38_extended.r”.
  b. eQTL.
    i. From each tissue type, extract only GTEx eQTL SNP results that match the GWAS SNP GRCh38 chromosome and base pair position. The output is an R object of GTEx eQTL for suggestively significant variants.
      See script: “GTEx.eQTL.all.assoc_extract_GWAS.sugg.SNPs.server.r”
    ii. Concatenate filtered GTEx eQTL files from each tissue corresponding to the GWAS suggestively significant variants. Iteratively load, annotate with tissue source, extract GRCh38 chromosome and base pair position from variant ID, and append each file into a single data frame in R.
    iii. Calculate the false discovery rate (FDR) per variant across all genes and all tissues.
    iv. Using the GTEx Ensembl IDs, map to gene symbol and Entrez IDs using the “EnsDb.Hsapiens.v86” package in R.
    v. Merge with Immune-Germline SNP annotation.
    vi. Extract GTEx eQTL results for variant-gene pairs with an FDR < 0.05 in at least one tissue. Exclude the HLA and IL17R locus which are simple eQTLs.
    vii. **Optional**: Visualize results by plotting the GTEx eQTL -log_10_ FDR p-value against the distance from the TSS (“tss_distance”).
      See scripts: “GTEx.eQTL.all.assoc_processResults_GWAS.sugg.SNPs_extended.r” and “GTEx.eQTL.all.assoc_processResults_GWAS.sugg.SNPs_1mb_extended_plot.r”.
  c. sQTL.
    i. From each tissue type, extract only GTEx sQTL SNP results that match the GWAS SNP GRCh38 chromosome and base pair position. The output is an R object of GTEx eQTL for suggestively significant variants.
    ii. Run the Linux bash script “run_split_sqtl.sh”. Each GTEx sQTL file is very large. This step separates the large GTEx sQTL file into a number of small files. The input of this script is a list of file names for GTEx sQTL. Each line of this input file is a file name for GTEx sQTL. The script generates a number of small files for each original GTEx sQTL file.
    iii. Run the R script “r_extract.txt”. This script takes 2 input files. One is an R object for GWAS suggestively significant variants. The other input file is the GTEx sQTL file generated from the previous step. The output is an R object of GTEx sQTL for suggestively significant variants.
    iv. Concatenate filtered GTEx sQTL files from each tissue corresponding to the GWAS suggestively significant variants. Iteratively load, annotate with tissue source, extract GRCh38 chromosome and base pair position from variant ID, and append each file into a single data frame in R.
    v. For sQTL, limit analysis to +/- 500 KB region. Filter the resulting concatenated GTEx sQTL file using the absolute value of the “tss_distance”, set a threshold ≤ 500,000 bp.
    vi. Calculate the false discovery rate (FDR) per variant across all genes and all tissues.
    vii. Using the GTEx Ensembl IDs, map to gene symbol and Entrez IDs using the “EnsDb.Hsapiens.v86” package in R.
    viii. Merge with Immune-Germline SNP annotation.
    ix. Extract GTEx sQTL results for variant-gene pairs with an FDR < 0.05 in at least one tissue. Exclude the HLA and IL17R locus which are simple eQTLs.
    x. **Optional**: Visualize results by plotting the GTEx sQTL -log_10_ FDR p-value against the distance from the TSS (“tss_distance”).
      See scripts: “GTEx.sQTL.all.assoc_processResults_GWAS.sugg.SNPs_500kb _extended.r” and “GTEx.sQTL.all.assoc_processResults_GWAS.sugg.SNPs_500kb_extended_plot”.
      ***Note:*** Scripts and code description used in this section are available at: https://github.com/rwsayaman/TCGA_PanCancer_Immune_Genetics.

Direct link: Expression and splicing quantitative trait locus analysis (eQTLs and sQTL). The example code in this section were optimized for the high-performance compute environment at UCSF HPC employing Portable Batch System (PBS) job scheduling; consult your system administrator to adapt the provided code to your system.

### Colocalization with eCaviar and manual curation of the expanded region

**Timing: 1 Week**

This section describes colocalization analysis performed with eCAVIAR. This analysis was performed using TCGA and GTEx gene expression data. This analysis requires four input files: (1) GWAS summary statistics, (2) eQTL summary statistics, (3) LD matrix computed with GWAS data, and (4) LD matrix computed with genotype data used for eQTL analysis.

33. TCGA Analysis.
  a. Create a file to determine SNP-gene-trait to be tested for colocalization. Run R script “DetermineRegions.R”. This script reads eQTL results and keeps SNP-gene eQTL FDR p < 0.1. Output of this script will be a file that contains 5 columns: chromosome, position, gene name, trait, and SNP significance (GW or suggestive).
  b. For each SNP-gene pair, run eQTL analysis between the SNP and the gene, and also between the 200 extra SNPs centered at the selected SNP and the gene. Use R script “RunEQTL.sh”. This script creates a folder for each SNP-gene pair. It extracts the list of 201 SNPs, calculates LD matrix (plink –bfile EXTRACTED –r square –out EXTRACTED), and performs GWAS and eQTL association analysis using the “eQTL.R” R script. “eQTL.R” script outputs two files: one for GWAS and one for eQTL analysis containing the z-score, beta, and p-value.
  c. Run eCAVIAR using “RunECAVIAR.sh” script. It calls eCAVIAR as follows:
    eCAVIAR -o coloc_c1.out -l EXTRACTED.ld -l EXTRACTED.ld -z GWAS.z -z eQTL.z -f 1 -c 1
    The “GWAS.z” and “eQTL.z” are the z-score produced in the previous step. “EXTRACTED.ld” is a 1-line file that contains the LD between the selected SNP and the 200 SNPs surrounding it (100 SNP to the left and 100 SNPs to the right). The -c flag indicates the number of causal variants assumed in the model (Model was run twice assuming 1 and 2 causal variants). The output that contains the colocalization posterior probability (CLPP) for each SNP (total of 201 SNPs) is “coloc_c1.out_col”.
  d. The same strategy is conducted for sQTL results.
34. GTEx Analysis.
  a. Run the R script “r_match_tcga_gtex.txt” to match the effect allele between GTEx eQTL/sQTL results and TCGA GWAS results. It then calculates Z scores for both GTEx eQTL/sQTL and TCGA GWAS results.
  b. This R script requires 4 input files:
    i. A table for index SNPs. The 1st column should be SNP ID and the 3rd column should be base-pair position in build 38.
    ii. A list of GTEx eQTL/sQTL results. Each line is the name of a GTEx eQTL/sQTL result. The last part of the file name should be “_rsid.txt”. Each GTEx eQTL/sQTL result file has the following columns: “gene_id”, “variant_id”, “tss_distance”, “ma_samples”, “ma_count”, “maf”, “pval_nominal”, “slope”, and “slope_se”.
    iii. A PLINK “.bim” file for TCGA genotype data. It has the following columns: chromosome, SNP ID, genetic distance, base-pair position, minor allele, and major allele.
    iv. A GWAS result file for TCGA. It has the following columns: chr:bp, CHR, SNP ID, BP, A1, A2, Genotyped, Rsq, AvgCall, MAF, Stratified.Freq, NCHROBSTEST, NMISS, BETA, STAT, and P.
  c. This R script generates 6 output files:
    i. Z score for GTEx eQTL/sQTL result.
    ii. Z score for TCGA GWAS result.
    iii. The list of SNPs in GTEx eQTL/sQTL result.
    iv. The list of SNPs in TCGA GWAS result.
    v. The list of SNPs and effect alleles in GTEx eQTL/sQTL result.
    vi. The list of SNPs and effect alleles in TCGA GWAS result.
  d. Output files iii-vi will be used to generate LD matrix for eCAVIAR. Output files i and ii will be used directly for eCAVIAR.
  e. Run PLINK commands to generate numeric genotype data for GTEx and TCGA for SNPs that were extracted as output files iii and iv from the previous step.
    i. Run the python script “make_plink_command_gtex.py” and “make_plink_command_tcga.py” to generate PLINK commands for GTEx and TCGA separately. The output is a Linux bash file that contains a number of PLINK commands.
    ii. Run the bash file from steps 34e–i.
      Example for GTEx:
      plink --bfile /wynton/scratch/dhu/gtex_geno/GTEx_WGS_chr11
      --extract snps_gtex_ENSG00000005801_Nerve_Tibial_ZNF195_rs7951724.txt --make-bed --out temp_gtex
      plink --bfile temp_gtex --a1-allele snps_alt_gtex_ENSG00000005801_Nerve_Tibial_ZNF195_rs7951724.txt 2 1 --recode A --out Geno_gtex_ENSG00000005801_Nerve_Tibial_ZNF195_rs7951724
      Example for TCGA:
      plink --bfile/wynton/scratch/dhu/tcga_geno/tcga_imputed_hrc1.1_noMissnp_b38_chr11 --extract snps_tcga_ENSG00000005801_Nerve_Tibial_ZNF195_rs7951724.txt --make-bed --out temp_tcga
      plink --bfile temp_tcga --a1-allele snps_alt_tcga_ENSG00000005801_Nerve_Tibial_ZNF195_rs7951724.txt 2 1 --recode A --out Geno_tcga_ENSG00000005801_Nerve_Tibial_ZNF195_rs7951724
  f. Run the R scripts “r_cor_gtex.txt” and “r_cor_tcga.txt” to calculate Pearson correlation coefficients for each pair of SNPs in GTEx and TCGA genotype data. There are 2 input files for each R script. The 1st one is the genotype file that was generated from the previous step. The 2nd one is the file with SNP ID and alternate allele. This file was generated as the output file 5 or 6 in the step for running “r_match_tcga_gtex.txt”.
  g. Run eCAVIAR assuming 2 causal SNPs.
    i. Run the python script “make_ecaviar.py” to generate commands to run eCAVIAR. The output is a Linux bash script that contains a number of eCAVIAR commands.
    ii. Run the Linux bash script from steps 34g–i.
      Example:
      eCAVIAR -l Cor_tcga_ENSG00000281491_Minor_Salivary_Gland_DNAJB5-AS1_rs72729406.txt -l Cor_gtex_ENSG00000281491_Minor_Salivary_Gland_DNAJB5-AS1_rs72729406.txt -z Result_tcga_ENSG00000281491_Minor_Salivary_Gland_DNAJB5-AS1_rs72729406.txt -z Result_gtex_ENSG00000281491_Minor_Salivary_Gland_DNAJB5-AS1_rs72729406.txt -c 2 -f 1 -o Result_eCAVIAR_c2_ENSG00000281491_Minor_Salivary_Gland_DNAJB5-AS1_rs72729406.txt
      ***Note:*** All programming scripts for GTEx colocalization were run on the UCSF Wynton HPC (https://wynton.ucsf.edu/hpc/) employing Portable Batch System (PBS) job scheduling. An example of script for submitting jobs on the cluster is “qsub_run_plink_gtex.txt”. The command for submitting the job is “qsub_run_plink_gtex.txt” and depends on the setup of the HPC cluster; consult your system administrator to adapt the provided code to your system.
      ***Note:*** Scripts and code description used in this section are available at: https://github.com/rwsayaman/TCGA_PanCancer_Immune_Genetics.

Direct link: Colocalization with eCaviar and manual curation of the expanded region.

### Rare variant analysis

**Timing: 1 week**

This section includes a workflow to assess the contribution of rare cancer predisposition variants on different immune traits.

35. Download VCF germline file (Huang et al., 2018) (see “key resources table”). Sub-select samples for which genotyping data are available and at least one phenotype is available (use “Sample List” as in “Table S1” from (Sayaman et al., 2021)).
36. Download annotations of curated Pathogenic and Likely Pathogenic Cancer Predisposition Variants from (see “Table S2”, (Huang et al., 2018)), which consists in 853 variants.
37. Annotate per-sample mutations using position information available in “Table S2” from (Huang et al., 2018).
  a. Extract unique variants from “Table S2” from (Huang et al., 2018) and write them in a space-delimited text file, say “list_snps.txt”, with format as follows: chromosome, position, position, dummy variable.
  b. Split downloaded vcf file per chromosome as follows, where “i” is the chromosome number:
    bcftools view -r $i PCA.r1.TCGAbarcode.merge.tnSwapCorrected.10389.vcf.gz -O b -o CHR"$i".bcf.gz
  c. Left normalize variants:
    bcftools norm -Ou -m -any CHR"$i".bcf.gz | bcftools norm -Ou -f human_g1k_v37.fasta | |bcftools --missing-to-ref | bcftools annotate -Ob -x ID -I +'%CHROM:%POS:-:%ALT' -O z -o CHR"$i"_norm.vcf.gz
  d. Replace missing genotypes by homozygous reference:
    bcftools +setGT CHR"$i"_norm.vcf.gz -O z -o CHR"$i"_norm_nomissing.vcf.gz -- -t . -n 0p
  e. Convert vcf files to PLINK formatted files:
    plink --vcf CHR"$i"_norm_nomissing.vcf.gz --keep-allele-order --vcf-idspace-to _ --const-fid --allow-extra-chr 0 --split-x 2699520 154931044 no-fail --make-bed --out CHR"$i"_norm_nomissing
  f. Extract the variant list prepared in step 37a and recode them mutations additively:
    plink --bfile CHR"$i"_norm --extract range list_snps.txt --recode A --out OUT$i --allow-no-sex
38. Collapse mutations into genes (i.e., consider as “gene event” if any pathogenic or likely pathogenic variant is present in that gene).
39. Collapse genes into mutually exclusive pathways (use category assignment as for in “Figure S7” from (Sayaman et al., 2021), which are called here “genotypic variables”.
40. Only retain genotypic variables with at least 5 events across cancer.
41. Download phenotypes as in “Table S2” from (Sayaman et al., 2021). Download genetic ancestry PC1-7, sex, age, and cancer type from “Table S1” from (Sayaman et al., 2021).
42. Annotate each sample according to MANTIS score from (Bonneville et al., 2017) (“Data Supplement S1”). Use 0.4 as cut-off for MSI-H bs MSI-S.
43. Run regression models pan cancer and per cancer for genes and pathways using “RareVariantAnalysis.R”.
44. Perform pan-cancer analysis.
  a. For phenotypes with normal/quasi-normal distribution (see “Table S2”, (Sayaman et al., 2021)), run linear regression between genotypic variables with at least 5 events per cancer, using as co-variates, genetic ancestry PC1-7, sex, age, and cancer type. For selected variables (see “Table S2”, (Sayaman et al., 2021)), apply Log Transformation.
  b. For phenotypes with skewed distribution, dichotomize values as low vs high (see “Table S2”, (Sayaman et al., 2021)), run logistic regression between genotypic variables with at least 5 events per cancer, using as co-variate genetic ancestry PC1-7, sex, and age.
45. Peform per-cancer analysis.
  a. Repeat steps 44a–b, without including cancer type as covariate.
46. Generate outcome summary: exome files related to samples for which all the covariates and at least one immune trait was available should result in a master file of N = 9,138 samples. There will be 832 pathogenic/likely pathogenic SNPs/Indels events with at least one copy of rare allele in the whole exome sequencing data, corresponding to 586 distinct pathogenic SNPs/Indels mapping to 99 genes. The regression analysis provides p-values and beta coefficients of the association with immune traits.

***Note:*** Scripts and code description used in this section are available at: https://github.com/rwsayaman/TCGA_PanCancer_Immune_Genetics.

Direct link: Rare Variant Analysis.

## Expected outcomes

The analysis protocols described above each yield data output files, as described above. Expected output files are summarized here. Data visualization tools enable researchers to explore these results interactively.

### Heritability analysis

Output files from GCTA GREML is an .hsq file. For complete description of output variables, see: https://cnsgenomics.com/software/gcta/#GREMLanalysis. The combined results table include the ratio of genetic variance to phenotypic variance (Vg/Vp) estimate and SE; the likelihood-ratio test (LRT) p-value and sample size (n) for each immune trait; and the FDR p-value across all immune traits.

After conducting heritability analysis across 139 immune traits, we identified 10 immune traits with significant heritability (FDR p < 0.05), and 23 other traits with nominally significant heritability (p < 0.05) in at least one ancestry group. Within the European ancestry group, 28 traits had at least nominally significant heritability (see “Table S3”, (Sayaman et al., 2021)).

### Genome-wide association study (GWAS)

The output from performing GWAS in PLINK consist of .assoc.linear (or .assoc.logistic) files with the following columns: chromosome code (CHR), variant identifier (SNP), base-pair coordinate (BP), allele 1 (A1), allele 2 (A2), test identifier (TEST) number of observations with nonmissing genotype, phenotype, and covariates (NMISS), regression coefficient (BETA for linear, or odds ratio OR for logistic), t-statistic (STAT) and asymptotic p-value for t-statistic (P). Note, running the PLINK command with parameter --keep-allele-order forces the original A1/A2 allele encoding and A1 should be the minor allele as originally encoded. PLINK output files are further described here: (https://www.cog-genomics.org/plink/1.9/formats#assoc_linear). After optional SNP annotation, addition columns include: the rsID and IUPAC nucleotide ambiguity codes from “SNPlocs.Hsapiens.dbSNP144.GRCh37”; the Genotyped, Rsq, AvgCall, MAF columns from Minimac3 HRC imputation information file; the recalculated MAF for the GWAS samples (n=9,603); nearest genes to SNP of interest (witing +/-50 KB, +/-500 KB, +/- 1,000 KB); VEP annotation.

After combining the GWAS summary stats of SNPs with genome-wide or suggestive significance from each of the 33 heritable immune traits, and annotating genome-wide significant loci, we identified 598 genome-wide significant (p < 5 × 10^−8^) associations at 23 loci for 10 immune traits. We also identified an additional 1,196 suggestive (p < 1 × 10^−6^) associations for 33 traits (see “Table S4”, (Sayaman et al., 2021)).

### Epigenomic analysis

The output table includes all genome-wide and suggestively significant SNPs annotated with the mapped epigenetic state from the Roadmap Epigenomics Project Expanded 18-state model. Each epigenome ID/epigenome represent a column with entries designating the epigenetic state (numeric and descriptive value) at the SNP chromosome and base pair position (see “Table S4”, (Sayaman et al., 2021)).

### Expression and splicing quantitative trait locus analysis (eQTLs and sQTL)

Output files for eQTL will have the following: chromosome, position, gene name, distance from gene, pan-cancer sample size, pan-cancer effect size, pan-cancer p-value, and then the same information repeated for each cancer type.

Output file for sQTL will contain association results as follows: chromosome, position, ensemble ID, gene name, splicing event ID.

### Colocalization with eCaviar and manual curation of the expanded region

Colocalization output table includes all genome-wide and suggestively significant SNPs and associated germline-immune GWAS summary stats from our study, the GTEx or TCGA QTL summary stats, and the eCaviar statistics which include: CLPP value for 1 causal SNP; regional CLPP value for 1 causal SNP; CLPP value for 2 causal SNP; regional CLPP value for 2 causal SNP; and the maximum CLPP value from 1 or 2 causal SNPs analysis or regional CLPP analysis. Finally, statistics from counter-evidence SNP colocalization analysis are included that gives the counter SNP’s ID, rsID, GTEx or TCGA QTL p-value, germline-immune GWAS p-value, and distance to the QTL TSS; the -log_10_ QTL p-value of the index SNP and counter SNP, and difference between these values (delta Counter SNP-Index SNP); and the curated expanded range colocalization evidence assessment (see “Table S5”, (Sayaman et al., 2021)).

### Rare variant analysis

Exome files related to samples for which all the covariates and at least one immune trait was available should result in a master file of N = 9,138 samples. There will be 832 pathogenic/likely pathogenic SNPs/Indels events with at least one copy of rare allele in the whole exome sequencing data, corresponding to 586 distinct pathogenic SNPs/Indels mapping to 99 genes. The regression analysis provides p-values and beta coefficients of the association with immune traits (see “Table S6”, (Sayaman et al., 2021)).

### Interactive visualization of results

**Figure 1:**
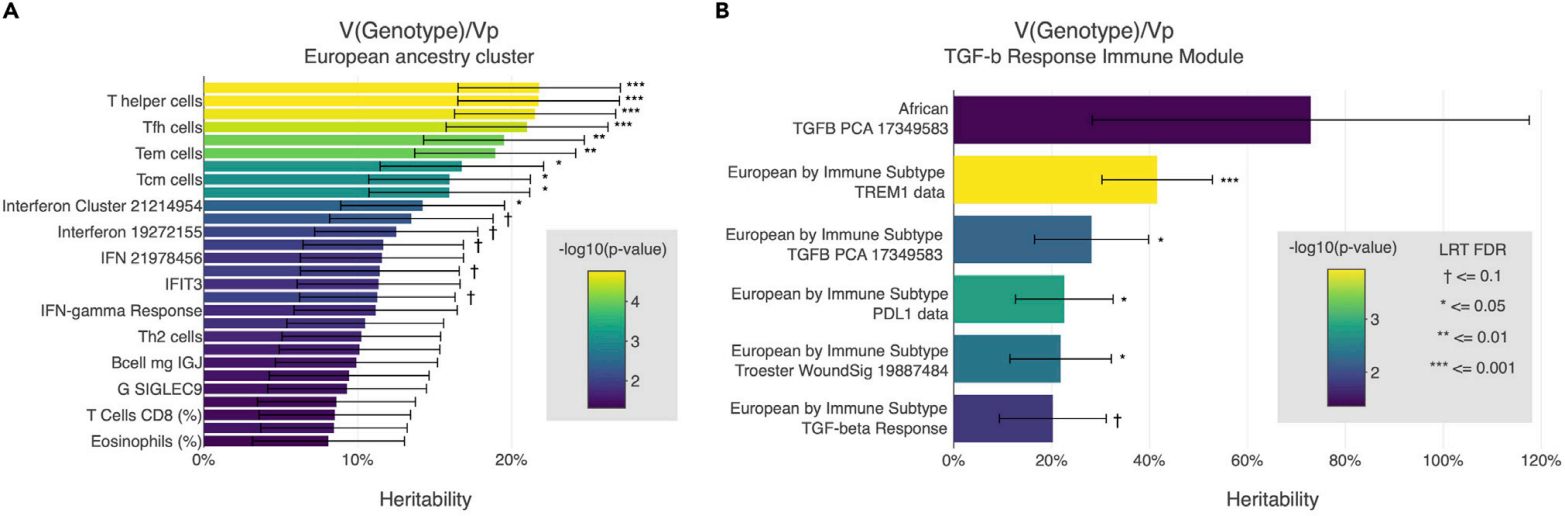
Visualization of heritability results in CRI iAtlas (A and B) (A) Figure 2A from the (Sayaman et al., 2021) manuscript can be reproduced, and (B) users can expand their analysis, by selecting to display results for selected immune features across different ancestry clusters, for example in the TGF-b Response module.

**Figure 2:**
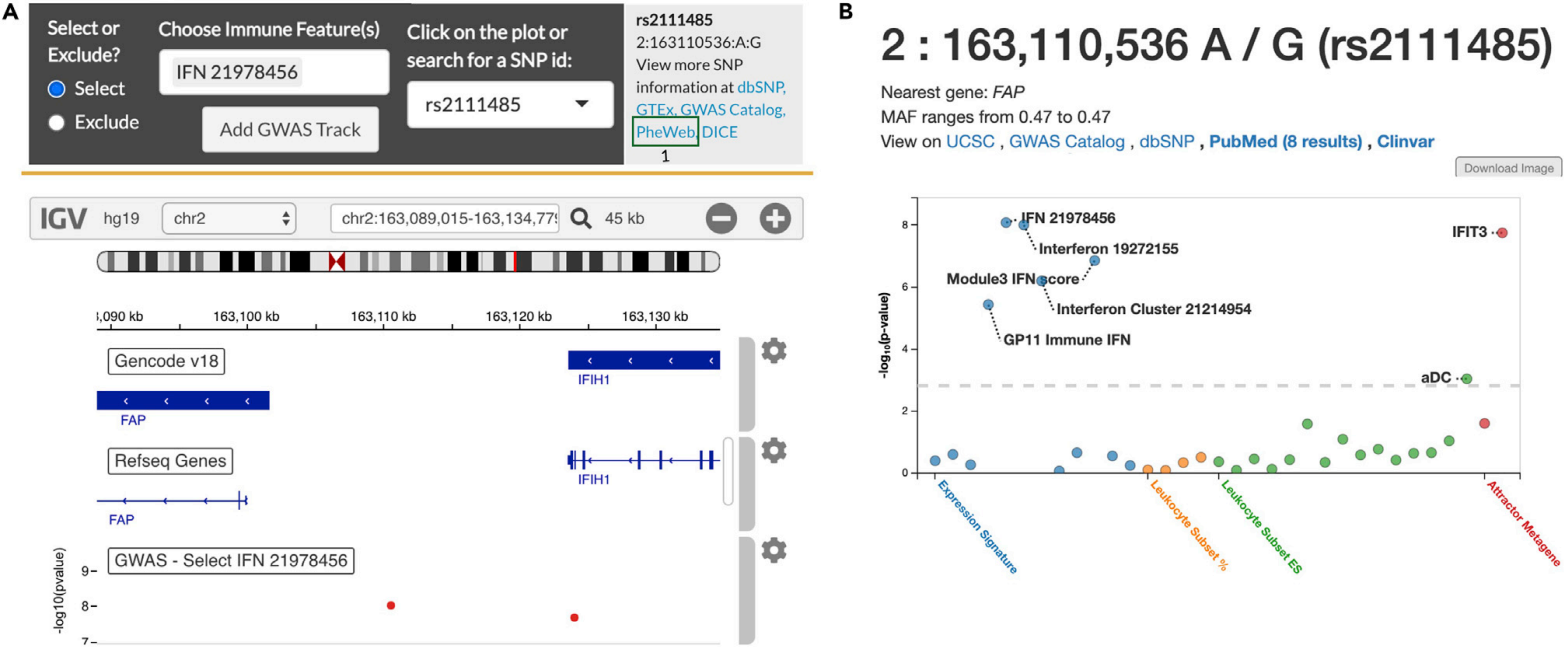
CRI iAtlas and PheWeb GWAS result visualization (A and B) Visualization of GWAS results in (A) CRI iAtlas and (B) PheWeb. (A) GWAS visualization in iAtlas consists of a Manhattan plot in an IGV track, accompanied by tracks with RefSeq Genes annotation and Gencode. Users can select a SNP of interest for a summary of GWAS hits associated with it, as well as links for external data sources for SNP information. By clicking on the PheWeb link (see green box labelled 1), users are redirected to (B) the corresponding SNP page in PheWeb.

**Figure 3:**
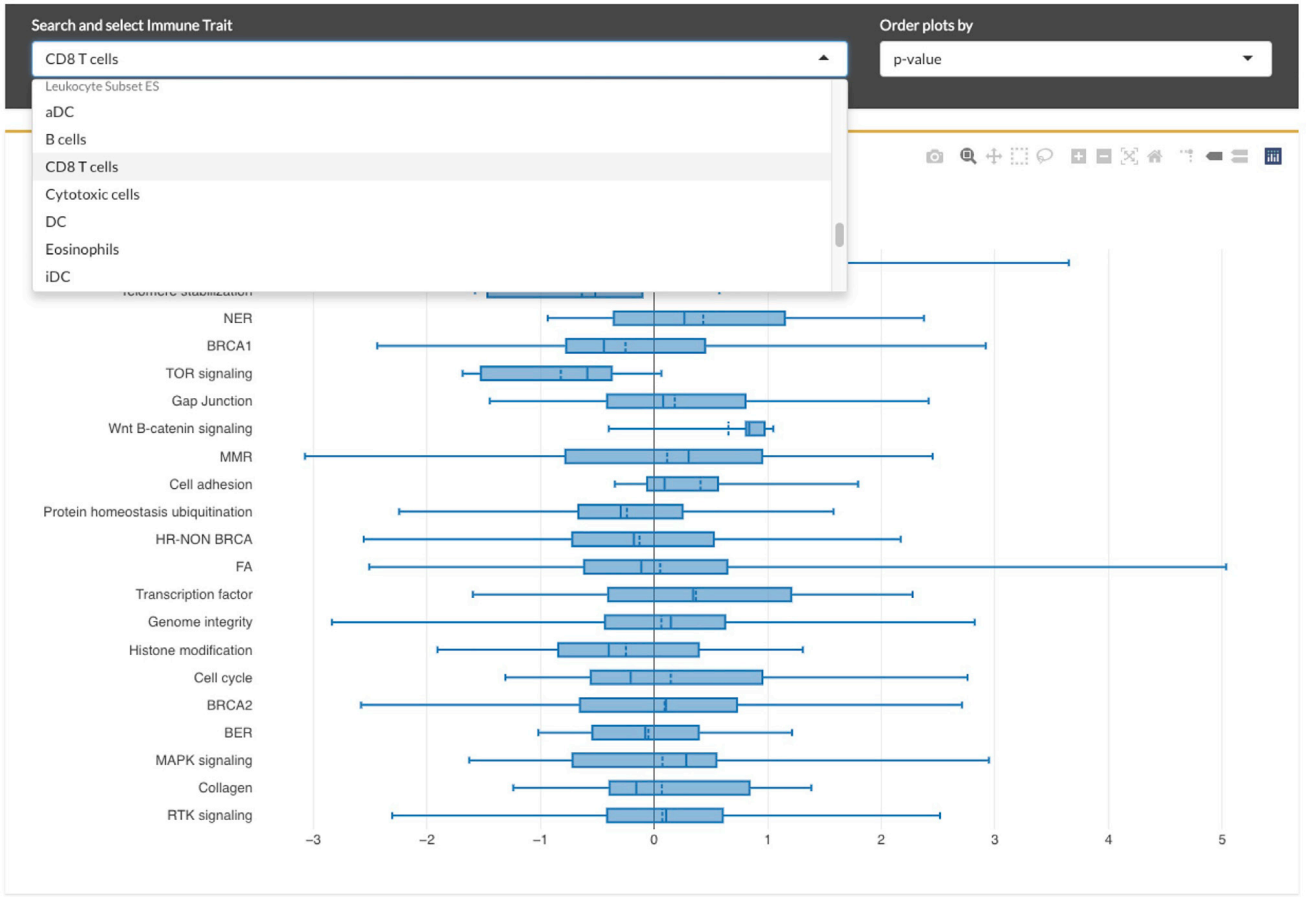
Visualization of rare variant analysis results in CRI iAtlas Users can select an immune feature of interest to see the distribution of its value across TCGA samples that have a mutation in a gene that is part of the listed pathways in the y-axis of the plot.

Results of the analysis can be explored interactively with web-based interactive visualizations. Results of heritability, GWAS, colocalization, and rare variant analyses can be visualized interactively in CRI iAtlas (https://www.cri-iatlas.org/), in the "Germline Analysis" module (Figures 1, 2, and 3, Methods video S1). CRI iAtlas is a web portal for data exploration of immuno-oncology research (Eddy et al., 2020). A summary of figures from the (Sayaman et al., 2021) manuscript that can be reproduced in CRI iAtlas is available in (Table 2). A PheWeb tool (https://pheweb-tcga.qcri.org/) was setup to visualize GWAS summary statistics of all tested immune traits (Figure 2B, Methods videos S2 and S3). Additional scripts for PheWeb GWAS initialization (Methods video S3) is provided at https://github.com/rwsayaman/TCGA_PanCancer_Immune_Genetics. Direct link: Interactive Visualization of Results.

Methods video S1. CRI iAtlas visualization tutorial, related to “Expected Outcomes: Interactive visualization of results” Overview of the interactive visualizations of heritability, GWAS and rare variant analysis results available in CRI iAtlas. https://figshare.com/s/ffae05886f705908afcb.

Method video S2. PheWeb GWAS visualization tutorial, related to “Expected Outcomes: Interactive visualization of results” Overview of the interactive visualizations of GWAS results available in PheWeb. https://figshare.com/s/4c12e5c52c175f73d16d.

Methods video S3. PheWeb GWAS initialization tutorial, related to “Expected Outcomes: Interactive visualization of results” Overview of the workflow used to generate PheWeb instance of GWAS results. https://figshare.com/s/ef620e57b95f254e6afb.

The CRI iAtlas "Germline Analysis" module (https://www.cri-iatlas.org/) is prepopulated with data and results from (Sayaman et al., 2021).

Heritability and GWAS results displayed in CRI iAtlas were collected from the (Sayaman et al., 2021) Supplementary Materials, respectively:

“Table S3. Genome-wide Heritability Results”.

“Table S4. Significant and Suggestive Genome-wide Association Results and Annotation”.

Colocalization plots in iAtlas were collected from Figshare:

GTEx expanded region analysis plots: https://doi.org/10.6084/m9.figshare.13089341.

TCGA expanded region analysis plots: https://doi.org/10.6084/m9.figshare.13090031.

TCGA three level plots: https://doi.org/10.6084/m9.figshare.13708720.v1.

Rare variant analysis boxplots in iAtlas are generated from summary statistics computed from the germline data.

Immune traits labels in CRI iAtlas used the annotation in the “Thorsson.TableS1.friendlyLabel” column from “Table S2. Annotations and Values of Immune Traits” in the Supplementary Materials from (Sayaman et al., 2021).

https://www.cri-iatlas.org/The heritability section of iAtlas provides visualization of heritability estimates and likelihood-ratio test (LRT) p-values. The GWAS section provides visualization of significant GWAS hits (p < 10^−6^), and colocalization results with colocalization posterior probability (CLPP) > 0.01. See tutorial (Methods video S1).

The PheWeb tool (https://pheweb-tcga.qcri.org/) visualizes GWAS summary statistics of all tested immune traits (Sayaman et al., 2021). The online tool displays an interactive Manhattan plot for each trait and a summary of the most significant SNPs. Zoom-in and –out options are available to get a closer look at genomic regions, where Linkage Disequilibrium (LD) and gene tracks are shown. See tutorial (Methods videos S2 and S3).

In PheWeb, gene tracks represent the physical start and end of a gene and does not imply any “causal” relationship with the SNP in question. Ancestry used to compute LD can be specified by the user (European, African, South Asian, etc.). SNP significance can be also shown for a specific SNP across all tested traits. External resources for SNPs can be accessed from PheWeb (e.g., GWAS Catalog, dbSNP, etc).

## Limitations

This protocol and related scripts are tailored for the analyses of matched genomic and immune trait datasets in TCGA but can be applied to other datasets with similar structures. The majority of analyzed immune traits are derived from gene expression data and can be adapted to other studies. Code used for the generation of immune traits from gene expression data are provided, and have been previously applied to RNA-sequencing (Thorsson et al., 2018) and microarray (Wolf et al., 2014) (Amara et al., 2017) data. Supplemental code for quality-control analysis, stranding and imputation of raw genotyping data from SNP arrays (Chambwe et al., 2022) are also made available.

Below we reported specific limitations related to each step of the protocol.

**Immune Traits:** Immune signatures lack cancer-specific cell type resolution. The majority of immune traits were calculated based on specific gene sets from expression data (RNA-sequencing) from bulk tissue to generate estimates of immune cell activation or abundance using different enrichment or deconvolution techniques. Caution should be exercised when interpreting results in the context of specific tumors or cell types. However, many of these signatures were validated in specific tissues and cancers via FACS sorting or immunohistochemistry/immunofluorescence imaging of immune populations.

Many of the immune signatures are highly correlated and not independent measures. Interpretation of results should be considered in context of functional modules defined as clusters of highly correlated signatures. Distribution of immune trait values in the dataset should be considered, and transformation of the data should be performed as needed to approximate a normally distributed set. Traits with a high fraction of zero values should be considered for dichotomization.

**Heritability Analysis:** For heritability estimates run via GCTA GREML, the GCTA FAQ (https://cnsgenomics.com/software/gcta/#FAQ) states that at least 3,160 samples from unrelated individuals are needed to get estimates with standard errors (SEs) down to 0.1 for common SNPs. Only the European ancestry group meets this criterion. Nonetheless, heritability estimates were run in the smaller sized ancestry groups with expectation of large SEs to provide preliminary analyses of immune traits in ancestry groups that are not well studied or sampled.

Heritability analysis takes into account only common variants. In this protocol, we used MAF > 1% as cut-off. Contribution of rare variants are not accounted for and may explain “missing" heritability.

**GWAS:** Linear regression assumes the residuals are normally distributed. Immune traits with skewed distributions were first log_10_ transformed, those assessed to have close to normal distribution were used as continuous variables. However, some immune traits remained with very skewed distributions due to a high fraction of 0 values, these traits were converted to binary 0 and 1 values based on the median value and logistic regression was performed instead (see “Table S2” (Sayaman et al., 2021)).

GWAS was run pan-cancer on the non-hematologic cancers in TCGA which vary in cohort size from 36 (CHOL) to 999 (BRCA). Results may be representative of the most common cancers in TCGA. Post hoc analysis evaluation of associations per cancer (forest plots) can provide insight in the directionality of the betas per cancer and identify potential outliers.

For GWAS, we used an imputation R^2^ ≥ 0.5 and MAF ≥ 0.5% as cut-offs for inclusion. The HRC panel (version 1) consists of 64,976 haplotypes at >39M SNPs constructed from 20 whole genome sequencing studies (McCarthy et al., 2016). HRC has been shown to provide accurate genotype imputation at MAF as low as 0.1%; however, HRC was constructed from studies of predominantly European ancestries and thus, may be limited for identifying rare variants, particularly in non-European ancestry populations. Moreover, the first version of the HRC panel did not to include small insertions and deletions (indels). We have made the TCGA QC unimputed dataset available as a resource which can be imputed to other reference panels such as the 1000 Genomes Project (1000 Genomes Project Consortium et al., 2015), which better capture indels, or the newest and largest reference dataset, NHLBI TOPMed (Taliun et al., 2021), which includes >50% non-European sequenced participants and may improve detection of rare and ancestry-specific genetic variants.

**Epigenomic Analysis:** Epigenomes are tissue- or cell line-specific. Due to cellular heterogeneity of tissue composition, epigenomes represent chromatin states of the bulk population. Interpretation of results should take into account the epigenomes of interest. A specific focus was given to immune-associated epigenomes in the analysis.

**eQTLs and sQTL:** GTEx data are derived from normal, tissue-specific samples; QTLs may capture features of normal tissue and those associated with certain tissue types. TCGA data are cancer-specific; QTLs may capture features associated only with specific cancers. Both GTEx and TCGA include expression from bulk tissues and so may miss eQTLs from cells that represent a small fraction of the tissue.

Our analyses used both TCGA germline and matched gene expression and alternative splicing data; and GTEx germline and matched gene expression and alternative splicing data. The rationale for using TCGA is that some loci may exert their effects via changes in expression in cancer tissue or in cells in the tumor microenvironment. The rationale for using GTEx is that some loci may exert their effects via changes in expression in normal tissue such as normal immune cells. However, care should be taken in comparing the tumor to normal eQTL results since the sample sizes are different and since the TCGA includes some surrounding normal tissue. Single cell RNA-seq is likely more robust for these types of analyses. Single cell RNA-seq is currently not available from TCGA or GTEx but in the future, as it becomes available, these eQTL analyses could be repeated to identify more variants colocalizing with certain genes and/or to determine cell-specific eQTLs. Thus, future studies with single cell analyses are likely needed to definitively characterize which cells these tissues are acting in. As eQTL and sQTL and colocalization in TCGA have been performed pan-cancer (GWAS was performed pan-cancer because of the sample size requirement for this analysis), per-cancer analysis might reveal additional cancer-specific hits, which might be compared with the ones in the related tissue. Because of the relatively limited number of samples available for each cancer type, per-cancer GWAS and colocalization should be preferentially performed by combining additional cancer-specific sources containing both phenotypic and genotypic data beyond TCGA.

**Colocalization:** Our protocol performed colocalization within a 200 SNP window (+/- 100 from the index SNP). In some cases, we observed that there were SNPs outside of that window that had better association with gene expression but weaker or no association with the immune trait. Therefore, we also performed a manual inspection of the entire locus (1 MB for eQTLs, 500 KB for sQTLs). We only performed this expanded region analysis of colocalization if there was plausible evidence of colocalization (eCAVIAR CLPP > 0.01) for the 200 SNP window. This expanded region analysis was intended to provide a more stringent criterion.

To perform these, we plotted the negative log_10_ p-value of association with the immune trait for each SNP in the region on the X-axis and the negative log_10_ p-value for association with the relevant gene expression (for eQTL analysis) or splicing event (for sQTL analysis). If we found additional SNPs outside of the 200 BP window and they demonstrated a stronger effect for association with gene expression or the sQTL and weaker association with the index SNP, we developed additional criteria for colocalization. If we identified one or more SNPs outside of the window that had a –log_10_ p-value with expression or splicing that was > 1.5 than the index SNP, we considered that as negative evidence for colocalization. If the SNP(s) with stronger evidence for eQTL or sQTL association had a –log_10_ p-value that was ≤1.5 compared with the index SNP, we considered the evidence for colocalization as “intermediate.” Finally, if we found no other SNP in the entire region with strong eQTL or sQTL that had a better p-value than the index SNP and the eCAVIAR results gave a posterior probability of colocalization of > 0.01, we considered the evidence to be “strong.”

While we used this manual second step in addition to eCAVIAR, an alternative approach would have been to use COLOC (Giambartolomei et al., 2014). This package has the disadvantage that linkage disequilibrium is not explicitly modeled (unlike eCAVIAR.).

Within the TCGA dataset, colocalization was performed on a pan-cancer level. This analysis can be conducted on each cancer type separately, assuming that GWAS and eQTL analyses are also performed for each cancer type separately. Low sample size can be a limiting factor for the per-cancer analysis.

Colocalization is based on gene-expression data from TCGA and GTEx but can also be performed in different datasets that might be available.

**Rare Variant Analysis:** In this analysis, we focused on variants occurring in cancer-predisposition genes, as previously annotated by (Huang et al., 2018), aggregated in categories summarizing different biological processes or function as described in (Sayaman et al., 2021) (except for *BRCA1* and *BRCA2*, for which sufficient number of events existed to treat these genes individually). Different aggregation in functional categories might be defined by the users. Heterogeneity of germline calls and batch effect prevented us to run a comprehensive exome-wide analysis (Rasnic et al., 2019). For variables with heavily skewed distribution, we dichotomized them based on median values. Alternatively, ordinal regression might be used.

For rare variants, we used germline pathogenic or likely pathogenic cancer predisposition variants (extracted from whole exome sequencing data) as previously defined (allele frequency in 1000 Genomes and ExAC (release r0.3.1) < 0.05%) (Huang et al., 2018).

## Troubleshooting

### Problem 1

Cannot load software or run scripts on the high-performance compute server. Implementation of provided GitHub code produces error (see “before you begin: software installation”, steps 4–9, and scripts referenced in the “step-by-step method details”).

### Potential solution

Consult your institution’s IT or compute cluster administrator for proper installation of necessary software including all needed libraries based on the high-performance compute environment. Ensure that the proper software versions, including all libraries and dependencies, are installed. Software implementation may be version specific, the versions used in the protocol are provided to ensure reproducibility.

Provided code should be considered as a guide. Adjust parameters based on cluster capabilities and specifications. Job submission scripts are dependent on the resource allocation management system. E.g., the provided GitHub codes for heritability analysis and GWAS were optimized for the high-performance compute environment at University of California, San Francisco employing Portable Batch System (PBS) job scheduling; consult your system administrator to adapt the provided code to your system.

• For troubleshooting of heritability analysis in GCTA GREML, see: https://cnsgenomics.com/software/gcta.
• For troubleshooting of GWAS in PLINK, see: https://www.cog-genomics.org/plink/.
• For issues with installation of CRI iAtlas, see troubleshooting guide on the software website: https://github.com/CRI-iAtlas/iatlas-app.

### Problem 2

Cannot access the controlled access TCGA data from the GDC Portal, or the QC and HRC Imputed genotyping data from GDC publication page associated with (Carrot-Zhang et al., 2020): https://gdc.cancer.gov/about-data/publications/CCG-AIM-2020 (see “before you begin: download datasets”, step 12).

### Potential solution

All TCGA germline data are controlled access. Ensure that you have proper authentication credentials and dbGaP authorization (see “before you begin: institutional permissions”, steps 1–3). Make sure to follow all steps required by the GDC to obtain controlled access data including having an institution account, authentication through eRA Commons and dbGaP authorization. Step-by-step instructions for obtaining access can be found here: https://gdc.cancer.gov/access-data/obtaining-access-controlled-data.

### Problem 3

In performing heritability analysis, filtering of individuals based on the genetic relatedness matrix is confounded by sample stratification based on genetic ancestry (see "step-by-step method details: heritability analysis", steps 8 and 9).

### Potential solution

Heritability analysis should be done within each genetic ancestry cluster as genetic ancestry creates population stratification.

### Problem 4

Heritability estimates do not converge (see “step-by-step method details: heritability analysis”, step 10).

### Potential solution

Heritability estimates do not converge when sample sizes are insufficient to build predictors; exclude groups with small samples sizes from analysis.

### Problem 5

The genomic coordinates were different in the original TCGA and GTEx data. TCGA data were in Genome Reference Consortium Human Build 37 (GRCh37) but the GTEx data were in Build 38 (GRCh38) (see "step-by-step method details: expression and splicing quantitative trait locus analysis (eQTLs and sQTL)", step 32, and "colocalization with eCaviar and manual curation of the expanded region", step 34).

### Potential solution

We converted the genomic coordinates in TCGA from GRCh37 to GRCh38 using liftOver (https://genome-store.ucsc.edu/) so these two data sets can be compared.

### Problem 6

In addition, genomic coordinates of some pathogenic variants in “Table S2” of (Huang et al., 2018) did not exactly match the WES vcf file positions (see "step-by-step method details: rare variant analysis", steps 36–37).

### Potential solution

To match the pathogenic variants with the WES vcf file, the start position of each variant (“Column D” in “Table S2” of Huang et al. (2018)) will be used with some changes as follows: subtract 1 for deletions, and use the start position as is for substitutions and insertions.

### Problem 7

During visualization, LD colors between SNPs are shown as gray (see "expected outcomes: interactive visualization of results").

### Potential solution

This means that LD information is not available, because of two reasons: (1) the SNP in question was not present in the 1000 Genomes Project data (1000 Genomes Project Consortium et al., 2015) which was used to compute LD, and (2) the reference and alternative alleles are swapped. For the first problem, no solution exists within the visualization tool. For the second problem, make sure reference and alternative alleles are concordant with the reference genome alleles.

## Resource availability

### Lead contact

Further information and requests for resources and reagents should be directed to and will be fulfilled by the lead contact, Rosalyn Sayaman, rwsayaman@gmail.com.

### Materials availability

This study did not generate new unique reagents.

## Data Availability

The TCGA Genome Wide SNP 6.0 birdseed genotyping data and clinical data can be found at the legacy archive of the GDC (https://portal.gdc.cancer.gov/legacy-archive). The quality-controlled and HRC imputed genotyping data were contributed towards ancestry analyses in (Carrot-Zhang et al., 2020) and are accessible at the associated GDC publication page (https://gdc.cancer.gov/about-data/publications/CCG-AIM-2020, see the Supplemental Data Files section: “TCGA QC HRC Imputed Genotyping Data used by the AIM AWG from (Sayaman et al., 2021)”). Please cite (Sayaman et al., 2021) and (Chambwe et al., 2022) when using the quality-controlled and HRC imputed genotyping data. Note, access to the TCGA original birdseed files and the pre-processed, quality-controlled and HRC imputed genotyping data generated for (Sayaman et al., 2021) requires GDC controlled access permission approval. Details for software availability are in the “key resources table”. The code generated during this study and the required supplemental tables from (Sayaman et al., 2021) have been deposited to GitHub (https://github.com/rwsayaman/TCGA_PanCancer_Immune_Genetics). Supplemental code for quality-control analysis, stranding and imputation of raw genotyping data from SNP arrays from (Chambwe et al., 2022) are also available at GitHub (https://github.com/rwsayaman/TCGA_PanCancer_Genotyping_Imputation). The summary statistics from the (Sayaman et al., 2021) GWAS have been deposited to FigShare (https://doi.org/10.6084/m9.figshare.13077920). Interactive visualization tools are available at CRI iAtlas (https://www.cri-iatlas.org/) and PheWeb https://pheweb-tcga.qcri.org.
